# A hypoxia-related signature for clinically predicting diagnosis, prognosis and immune microenvironment of hepatocellular carcinoma patients

**DOI:** 10.1186/s12967-020-02492-9

**Published:** 2020-09-04

**Authors:** Baohui Zhang, Bufu Tang, Jianyao Gao, Jiatong Li, Lingming Kong, Ling Qin

**Affiliations:** 1 Department of Physiology, School of Life Science, China Medical University, No. 77 Puhe Road, Shenyang North New AreaLiaoning Province, Shenyang, 110122 People’s Republic of China; 2 Department of Radiology, School of Medicine, Second Affiliated Hospital, Zhejiang University, Hangzhou, 310058 China; 3 Department of Radiation Oncology, the First Affiliated Hospital of China Medical University, Shenyang, China; 4 Department of Orthopedics, The First Affiliated Hospital of China Medical University, Shenyang, 110001 Liaoning People’s Republic of China; 5 Department of General Surgery, Shengjing Hospital of China Medical University, Shenyang, 110004 China

**Keywords:** Hypoxia, Hepatocellular carcinoma, Prognostic, Diagnostic, Immune microenvironment

## Abstract

**Background:**

Hypoxia plays an indispensable role in the development of hepatocellular carcinoma (HCC). However, there are few studies on the application of hypoxia molecules in the prognosis predicting of HCC. We aim to identify the hypoxia-related genes in HCC and construct reliable models for diagnosis, prognosis and recurrence of HCC patients as well as exploring the potential mechanism.

**Methods:**

Differentially expressed genes (DEGs) analysis was performed using The Cancer Genome Atlas (TCGA) and Gene Expression Omnibus (GEO) database and four clusters were determined by a consistent clustering analysis. Three DEGs closely related to overall survival (OS) were identified using Cox regression and LASSO analysis. Then the hypoxia-related signature was developed and validated in TCGA and International Cancer Genome Consortium (ICGC) database. The Gene Set Enrichment Analysis (GSEA) was performed to explore signaling pathways regulated by the signature. CIBERSORT was used for estimating the fractions of immune cell types.

**Results:**

A total of 397 hypoxia-related DEGs in HCC were detected and three genes (PDSS1, CDCA8 and SLC7A11) among them were selected to construct a prognosis, recurrence and diagnosis model. Then patients were divided into high- and low-risk groups. Our hypoxia-related signature was significantly associated with worse prognosis and higher recurrence rate. The diagnostic model also accurately distinguished HCC from normal samples and nodules. Furthermore, the hypoxia-related signature could positively regulate immune response. Meanwhile, the high-risk group had higher fractions of macrophages, B memory cells and follicle-helper T cells, and exhibited higher expression of immunocheckpoints such as PD1and PDL1.

**Conclusions:**

Altogether, our study showed that hypoxia-related signature is a potential biomarker for diagnosis, prognosis and recurrence of HCC, and it provided an immunological perspective for developing personalized therapies.

## Background

Hepatocellular carcinoma (HCC) accounts for 85% of liver cancers, and the disease burden of HCC is increasing globally [1]. Although progress on treatment strategies for HCC has been made, the overall 5-year survival rate for HCC patients remains less than 20% [2]. Nowadays, the research of molecular mechanism based on bioinformatics analysis has become one of the most important tools for cancer research [3, 4]. Therefore, it is of great significance to search for molecular markers for early diagnosis, survival prediction and recurrence monitoring of HCC, which can improve patients’ stratification and optimize medical intervention. The low rate of early diagnosis and high rate of metastasis and recurrence have considerable impact on the prognosis of HCC patients, which are mainly related to the invasiveness and high proliferative activity of tumor cells [5]. However, the mechanism of tumor progression has not been completely realized.

Hypoxia is an intrinsic characteristic of solid tumors due to the imbalance between the rate of tumor cell proliferation and nutrient supply of vascular [6]. Existing studies have recognized the critical roles played by hypoxia on tumor angiogenesis, cell proliferation, as well as cell differentiation and apoptosis [7, 8]; Liver is one of the three organs most susceptible to hypoxia and it has been found that hypoxia was involved in the metastasis, poor prognosis and radiation resistance of HCC [9, 10]. Nevertheless, its potential regulatory mechanism remains unclear. In recent years, there is an increasing interest in the tumor microenvironment which immune cells in it play a crucial role in the progression of tumor [11–13]. Previous studies have shown that hypoxia can regulate the status of tumor immune microenvironment, such as promoting the recruitment of innate immune cells and interfering with the differentiation and function of adaptive immune cells [14]. Therefore, further study on the relationship between hypoxia and immunity in HCC is required in order to develop new therapeutic strategies.

Immunocheckpoint inhibition has become an effective and frequently-used way of immunotherapy [15]. As a new feature of cancer, tumor mutation burden (TMB) is defined as the total number of somatic mutations in the genome of tumor cells [16], and high TMB may produce many neoantigens to stimulate the anti-tumor immune response [16]. Clinical data demonstrated that patients with high TMB were more likely to benefit from immunocheckpoint inhibitor therapy [17, 18], which suggesting that TMB should be an appropriate biomarker for assessing the effect of immune treatment.

In this study, we analyzed hypoxia-related genes in HCC by using TCGA and GEO database and constructed a consistent clustering. Then we built the prediction model for diagnosis, recurrence and prognosis of HCC. We also explored the association of hypoxia with immune infiltration and immunocheckpoints in HCC. These findings may make a meaningful contribution to the development of comprehensive therapeutic strategies for HCC patients.

## Methods

### Identification of differentially expressedgenes (DEGs) between HCC and noncancer tissues

The differentially expressed genes (DEGs) related to hypoxia and HCC were identified with limma, an R package [19]. The DEGs with an absolute log2-fold change (FC) > 1 and an adjusted P value < 0.05 were considered for further analysis.

### Acquisition of hypoxia-related genes associated with HCC

The mRNA expression profiles and corresponding clinical information associated with HCC patients were obtained from The Cancer Genome Atlas—Liver Hepatocellular Carcinoma dataset (TCGA‐LIHC) (including 370 HCC and 50 normal tissue samples). The mRNA-sequencing data of Human HCC cell lines were obtained from the Gene Expression Omnibus database (GEO), which included GSE59729 (with gene expression profiles of Huh-7 cells under normoxia and hypoxia for 24 h) and GSE41666 (with gene expression profiles of HepG2 cells exposed to normoxia and hypoxia for 24 h). A total of 1,401 hypoxia-related DEGs expressed by HepG2 from GSE41666 and 1,279 hypoxia-related DEGs expressed by Huh7 from GSE59729 were matched with HCC-related information obtained from TCGA. The data from TCGA and GEO databases are freely available to the public, and this research also strictly followed access policies and publication guidelines, therefore this study did not require ethical review and approval from an Ethics Committee.

### Classification of molecular subgroups by consistent clustering

The ConsensusClusterPlus package in R software was utilized for the consistent clustering to determine subgroups of HCC samples from TCGA. The Euclidean squared distance metric and the K-means clustering algorithm was used for classifying samples into k clusters with k = 2 to k = 9. About 80% of the samples were selected in each iteration, and the results were compiled over 100 iterations. The results are presented in the form of heatmaps of the consistency matrix generated by pheatmap R package, and the optimal number of clusters was determined by the consistent cumulative distribution function (CDF) graph and the delta region graph [20]. We considered that the optimal number of clusters should satisfy the following criteria: high consistency of clustering, low coefficient of variation, and no significant increase in the area under the CDF curve. According to the relative non-significant change of the area under the CDF curve, the corresponding number of categories was determined.

### Establishment and validation of a prognostic predictive signature

The univariate Cox regression analysis was conducted to identify the prognostic value of the DEGs for OS and genes with a P value < 0.05 were considered statistically significant. Subsequently the Least absolute shrinkage and selection operator (LASSO) Cox regression [21] was performed by using the glmnet R package to shrink scope of gene screening, we performed 1,000 substitution samples of the dataset and selected the markers with repeat occurrence frequencies of more than 900. Finally, a multivariate Cox regression analysis was performed to identify highly correlated genes and construct the prognostic gene signature. The regression coefficient (β) was derived from multivariate Cox regression analysis and the Prognosis Index (PI) = (β_mRNA1_* expression level of mRNA_1_) + (β_mRNA2_* expression level of mRNA_2_) + … + (β_mRNAn_* expression level of mRNA_n_). Based on the optimal cut-off value determined by using X-tile software, patients with survival data were divided into high- and low-risk groups. The Kaplan–Meier survival analysis was used to evaluate the predictive ability of the prognostic model, which was further validated in the ICGC dataset.

### Independence of the prognostic gene signature from other clinical characteristics

Univariate and multivariate Cox proportional hazard regression analyses were performed to determine whether the predictive ability of prognostic model was independent of conventional clinical characteristics. A bilateral P value < 0.05 was considered statistically significant. The hazard ratio (HR) and 95% confidence intervals were calculated.

### Construction and evaluation of a predictive nomogram

All independent prognostic factors were used to build a nomogram [22] in order to evaluate the 1-, 3-, and 5-year survival probability for patients with HCC. The calibration plot was performed for an internal validation to verify the accuracy. Time-dependent receiver operating characteristic (ROC) analysis was conducted to evaluate the predictive performance of the nomogram. Decision curve analysis (DCA) was performed to assess the clinical net benefit [23].

### Gene set enrichment analysis

Gene set enrichment analysis (GSEA) [24] was performed using prognosis index with Clusterprofiler package to identify signaling pathways regulated by the hypoxia-related signature. The correlation coefficients, CI and P-values were calculated using R software. P < 0.05 was considered statistically significant.

### Estimation of immune cell type fractions

CIBERSORT is a method for characterizing the cell composition from their gene expression profiles and is the most frequently cited tool for estimating and analyzing immune cells infiltration [25]. We utilized CIBERSORT to estimate the fractions of immune cell types between low- and high-risk groups. The sum of all the estimated immune cell type scores is equal to 1 in each sample.

### Real-time PCR analysis

Total RNA was isolated using Trizol reagent (Invitrogen, Eugene, OR, USA). The first-strand.

cDNA was synthesized with Prime-Script RT Master Mix (TaKaRa) followed by qPCR detection using the SYBR Green Master Mix (TaKaRa). The following primers were used: PDSS1 F: 5′-AGCCAACAGTTGTAAAGCAGTATTT-3′ and R: 5′-GTTTGTTGCACACCATCACTCTGT-3′; CDCA8 F: 5′-GCACAGCGAGGTTTTGCTCA-3′ and R: 5′-AACTGGGTAGGGACGAGGA-3′; and SLC7A11 F: 5′-ATGGGACAAGAAACCCAGGTG-3′ and R: 5′-TCCCTATTTTGTGTCTCCCCTTG-3′.

### Statistical analysis

Continuous variables were summarized as the mean ± standard deviation (SD). Differences between groups were compared by Wilcox test through R software. Different hypoxia subtypes were compared by using the Kruskal–Wallis test. The significance of survival time differences was calculated using the log-rank test with a threshold of P-value < 0.05. Kaplan Meier curves were plotted to show the survival time differences.

## Results

### Identification of DEGs related to hypoxia in HCC

**Fig. 1:**
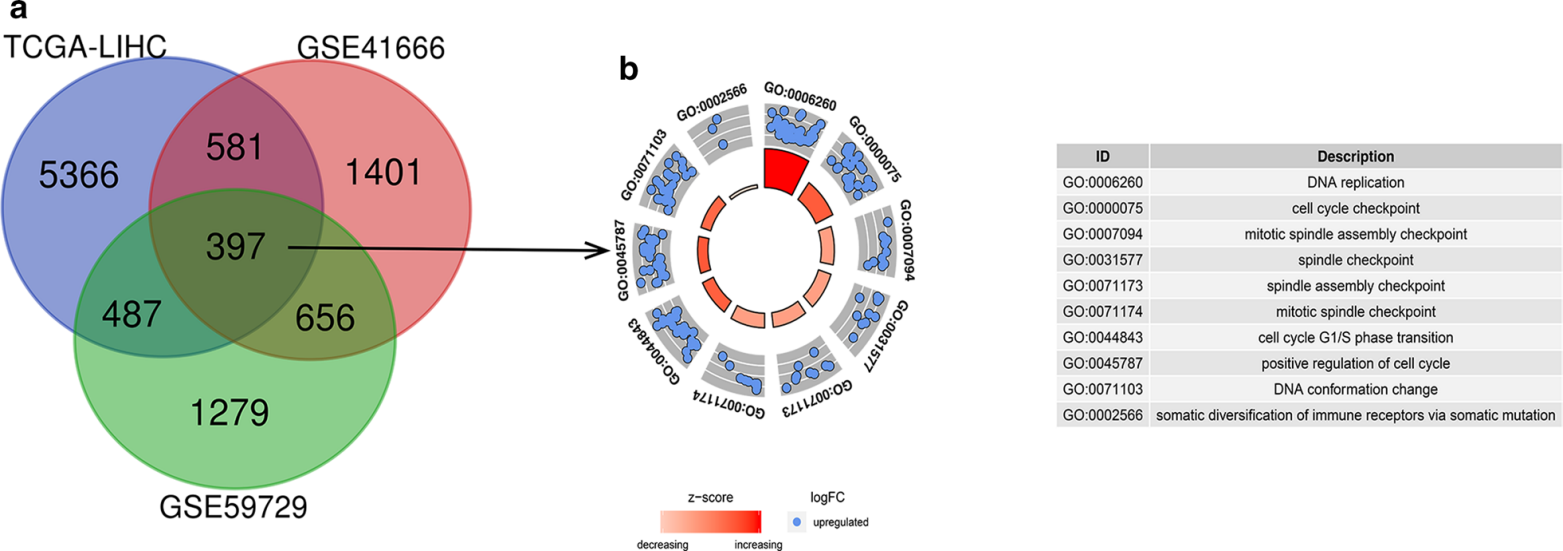
Volcano plot showing the differentially expressed hypoxia-related genes of HCC in different databases. A Common differentially expressed genes between the TCGA and GEO databases. B Gene Ontology (GO) analysis of 397 hypoxia-related DEGs in HCC

We identified DEGs (|LogFC|> 1, P < 0.05) using the mRNA expression profile between HCC and adjacent noncancerous tissues from TCGA database (Additional file 1: Table S1). Then we matched the differentially expressed mRNA-sequencing data between hypoxia-treated and untreated HCC cell lines in GEO database (Additional file 2: Table S2, Additional file 3: Table S3) and obtained 397 DEGs which were related to hypoxia in HCC (Fig. 1a). By using the Gene Ontology (GO) enrichment and functional analysis, we found that these genes are enriched in DNA replication, cell division, cell cycle and also somatic diversification of immune receptors (Fig. 1b).

### Using the hypoxia-related genes for the consistent clustering of HCC molecular subgroups

**Fig. 2:**
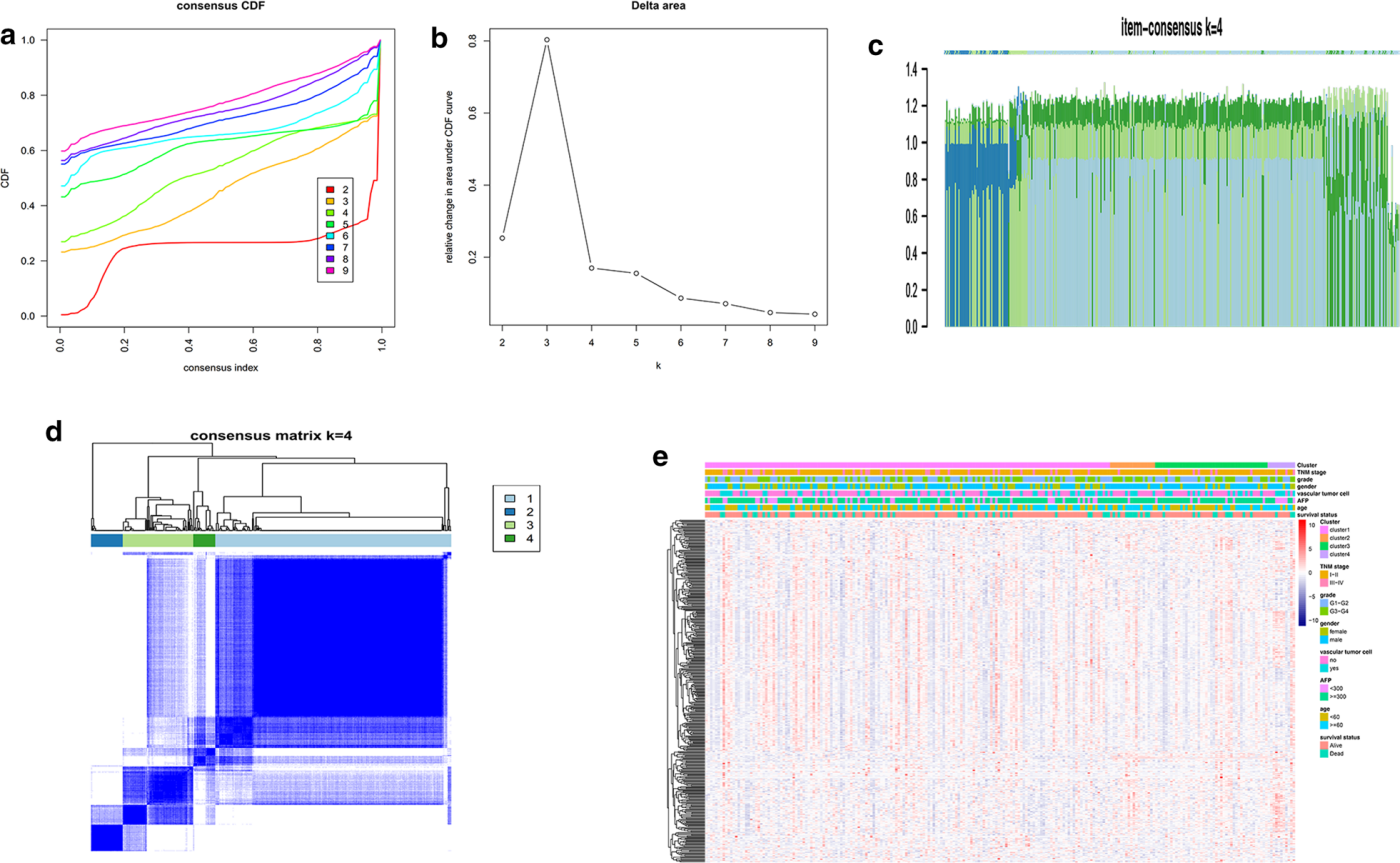
Consensus clustering of HCC molecular subgroups based on hypoxia-related DEGs. **a** Cumulative distribution function (CDF) curve. **b** CDF Delta area curve, which indicates the relative change in the area under the CDF curve for each category number k compared with k-1. The horizontal axis represents the number k and the vertical axis represents the relative change in the area under the CDF curve. **c** The Item-Consensus Plot for k = 4. The vertical axis represents item-consensus values and each bar represents each sample. **d** The heatmap corresponding to the consensus matrix for k = 4 obtained by applying consensus clustering. The rows and columns of the matrix represent samples. The values of the consistency matrix are shown in white to dark blue from 0 to 1, which represent the degree of consensus. **e** The heatmap of 397 hypoxia-related genes in 4 clusters

Consistent clustering of 397 hypoxia-related DEGs were constructed by using the ConsensusClusterPlus R software package. The average clustering consistency and inter-cluster variation coefficient of each cluster number were calculated and the optimal cluster number was determined by using CDF. As shown in Fig. 2a, the clustering outcoming was stable when k = 4. We further analyzed CDF delta area curve and found that the area under the CDF curve tended to be stable after 4 clusters (Fig. 2b). The item-Consensus Plot also showed that the sample classification was relatively stable when the clustering number was selected as 4 (Fig. 2c). Finally, we built a consensus matrix graph which 397 DEGs were assigned to 4 clusters in order to evaluate the composition and quantity of clustering more intuitively (Fig. 2d). The heatmap of 397 hypoxia-related DEGs in 4 clusters was shown in Fig. 2e.

**Fig. 3:**
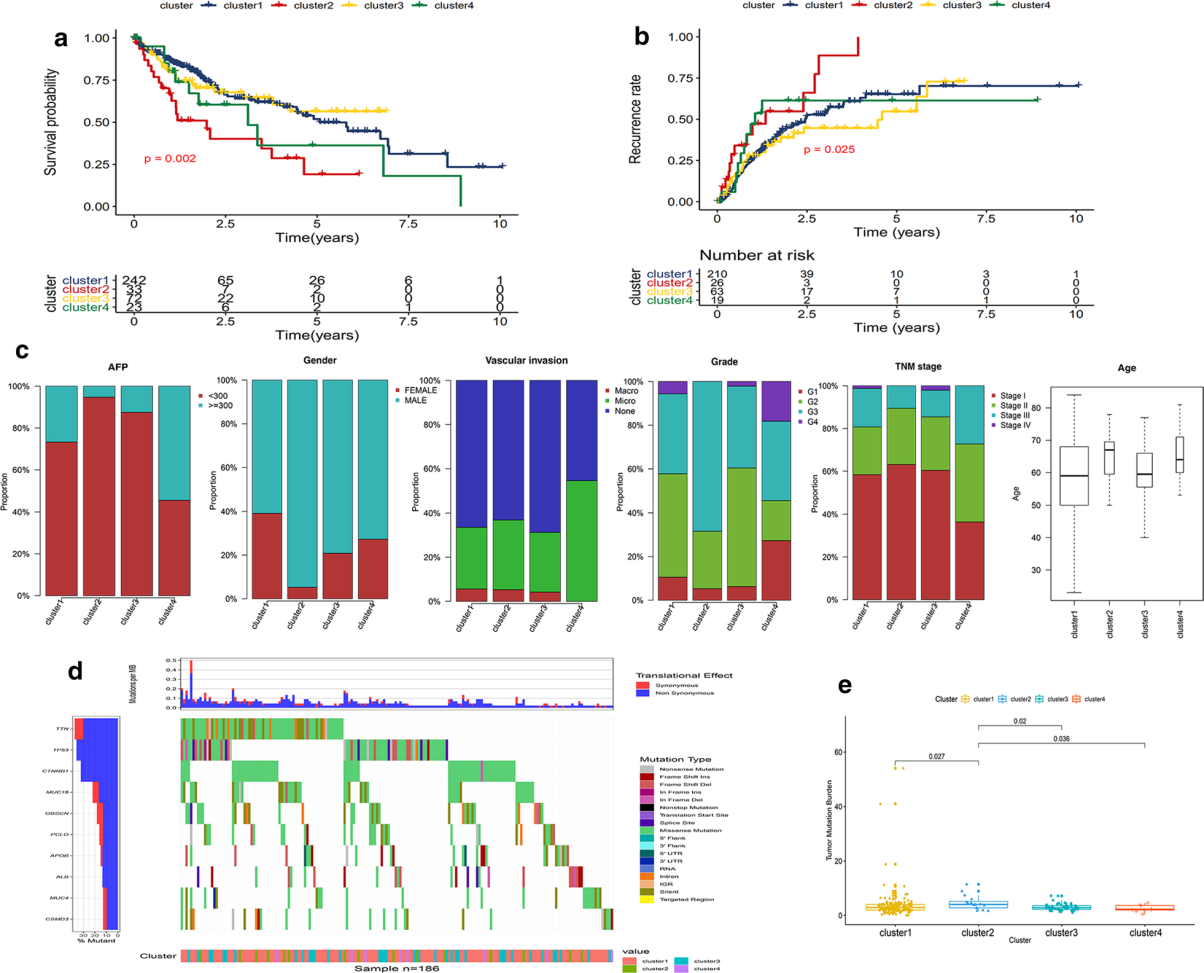
Characterization of different features of hypoxia-related DEGs clustering. **a**, **b** K‐M survival curves showed the differences of overall survival and recurrence rate among the 4 clusters. **c** Proportion of other clinical characteristics in 4 clusters. **d**, **e** The differences of TMB among 4 clusters

The results from Kaplan–Meier plot showed the significant differences in survival probability and recurrence rate among these 4 subgroups. Compared to the other three clusters, the samples in cluster-2 had the worst prognosis and the highest recurrence rate (Fig. 3a, b). We further analyzed the distribution of AFP, gender, degree of vascular infiltration, TNM stage, pathological grade, and age in these 4 subgroups (Fig. 3c). Samples in cluster-4 were associated with high AFP expression level, undifferentiated tumor cells and lymphatic metastasis while cluster-3 showed high incidence of distant metastasis; cluster-2 had a higher degree of vascular invasion and more tumor cells with low differentiation. Moreover, most of patients in cluster-2 were male and generally aged between 65 and 70 years. It is worth noting that patients in cluster-2 showed the highest TMB than other three clusters (Fig. 3d, e), suggesting a benefit of immunotherapy.

### Construction and validation of a hypoxia-related prognosis signature with good performance

**Fig. 4:**
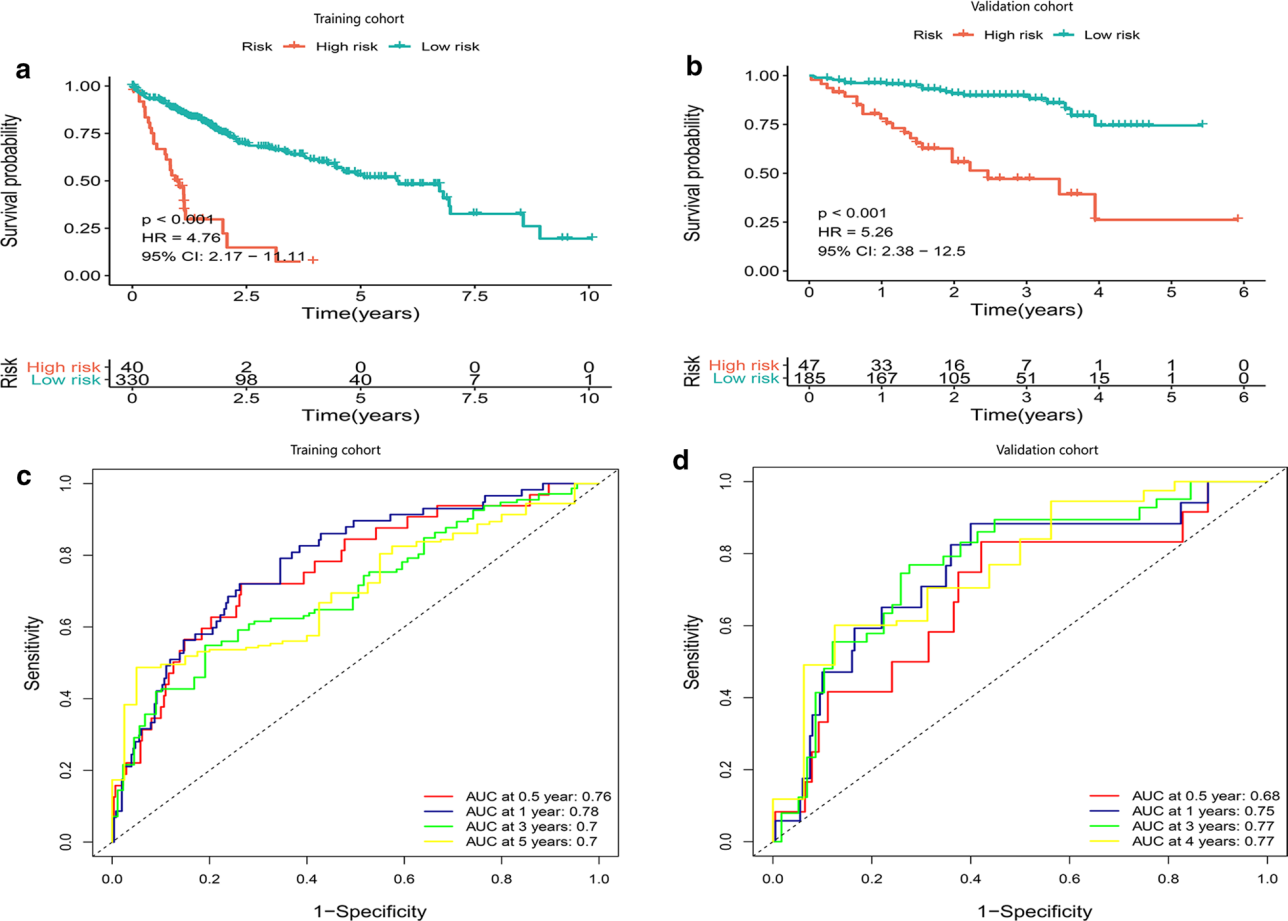
Kaplan–Meier analysis, risk score analysis, time‐dependent ROC analysis for a prognosis model based the three‐gene signature in HCC. **a**, **b** K–M survival curve of high- and low-risk in TCGA cohort and ICGC cohort. **c**, **d** Time‐dependent ROC analysis for OS prediction in TCGA and ICGC cohort

We performed a univariate Cox regression and found 291 DEGs significantly related to OS of HCC patients (P < 0.01) (Additional file 4: Table S4). Then a Lasso‐penalized Cox analysis was performed to further shrink the scope of gene screening. The penalty parameter was established through 10-fold cross‐validation. We selected 11 DEGs, which appeared over 900 times of a total of 1000 repetitions (Additional file 5: Figure S1). Finally, by analyzing a multivariate Cox regression, three genes (PDSS1, SLC7A11, CDCA8) conforming to the proportional hazards (PH) assumption were selected to build a prognostic model as follows: the prognostic index (PI) = (0.337 * expression level of PDSS1) + (0.383* expression level of SLC7A11) + (0.356* expression level of CDCA8). The optimal cut-off value of 2.296 for the risk store was produced using X-tile software and patients with survival time from TCGA-LIHC were divided into a high- and low-risk group. The K-M curve showed that the OS of the high-risk group was significantly poorer than that of low-risk group (P < 0.001, HR = 4.76) (Fig. 4a). The area under the time-dependent ROC curves (AUCs) for 0.5-, 1‐, 3‐ and 5‐year overall survival (OS) were 0.76, 0.78, 0.7 and 0.7, respectively, indicating a good predictive performance of this prognostic model (Fig. 4c).

We further validated the prediction ability of this prognostic signature using HCC samples from ICGC database (Additional file 6: Table S5). Consistent with above results, HCC patients were divided into a high- and low-risk group with an optimal cut-off value of 18.812 and patients in the high-risk group had poorer survival probability than the low-risk group (P < 0.001, HR = 5.26) (Fig. 4b). The AUCs of the three‐gene prognostic model were 0.68, 0. 75, 0.77 and 0.77 for the 0.5-, 1-, 3- and 4‐year survival times (Fig. 4d). Meanwhile, we attempted to compare the hypoxia-related signature with other prognostic models published previously [26, 27]. For the hypoxia-related signature, methylation-driven prognostic model and three-gene prognostic model, the AUCs was 0.78, 0.67 and 0.67 in TCGA cohort and 0.75, 0.64 and 0.64 in ICGC cohort, respectively (Additional file 7: Figure S2). Taken together, our prognostic model showed a higher specificity and sensitivity.

### Evaluating the independent role of prognostic signature and building a predictive nomogram for OS prediction in the HCC cohort from TCGA

**Fig. 5:**
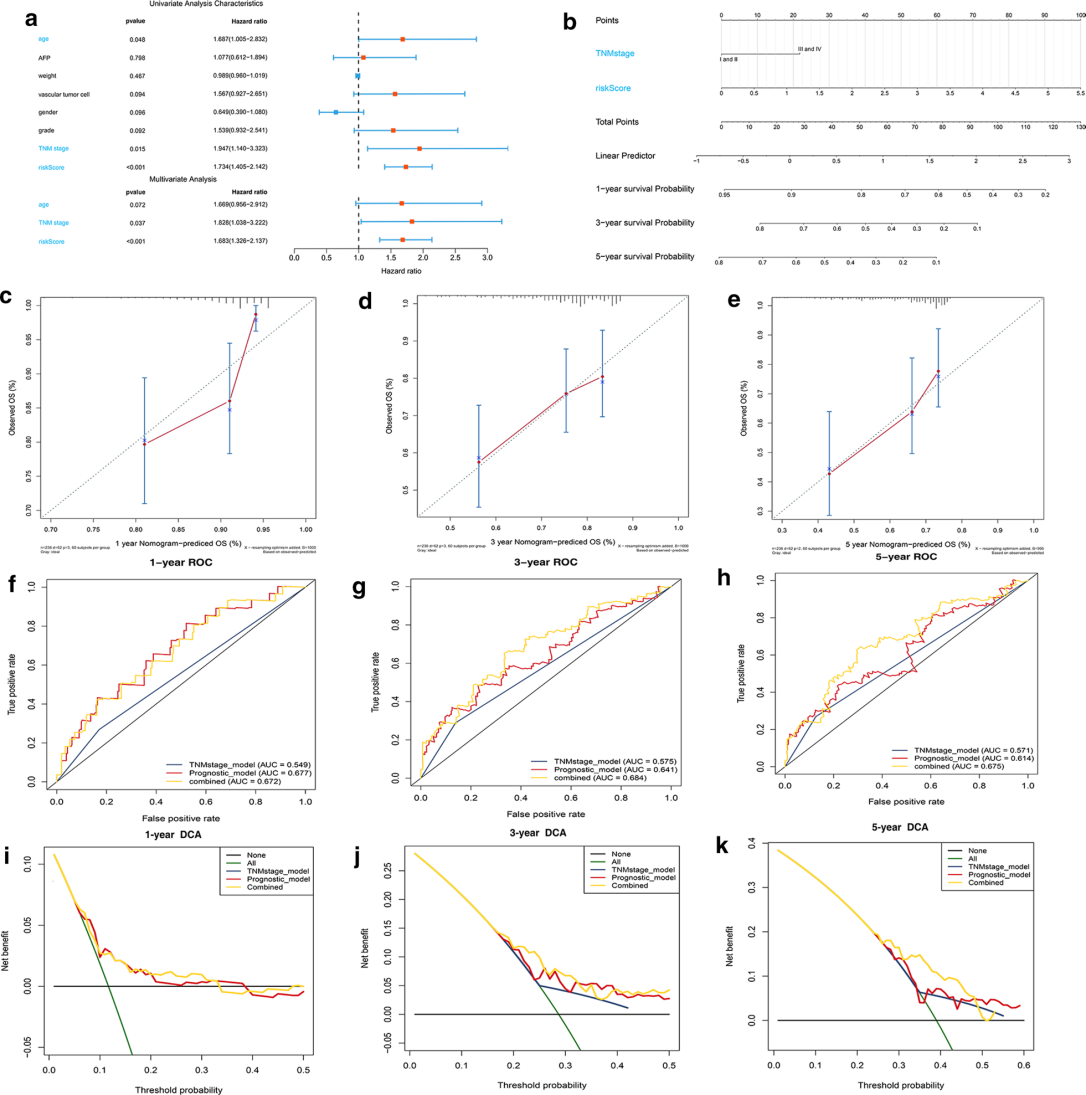
Construction of the nomogram predicting overall survival for HCC patients in the TCGA cohort. **a** Forrest plot of the univariate and multivariate association of the prognostic model and clinicopathological characteristics with overall survival. **b** The nomogram was built based on two independent prognostic factors for predicting OS in HCC patients at 1-, 3-, and 5-year. **c**–**e** The calibration plot for internal validation of the nomogram. **f**–**h** Time‐dependent ROC curves of the nomogram for 1‐,3‐ and 5‐year overall survival in HCC to evaluate the predictive performance of the nomogram. **i**–**k** DCA curves of the nomogram for 1‐,3‐ and 5‐year overall survival in HCC to evaluate the clinical decision-making benefits of the nomogram

Univariate and multivariate Cox regression analysis were used to evaluate whether the predictive value of the prognostic model was independent of other traditional clinical characteristics. The results showed that the TNM stage (P < 0.05, HR = 1.828) and the risk score (P < 0.05, HR = 1.683) were independent prognostic factors for OS (Fig. 5a). Then we built a predictive nomogram which may be helpful to accurately predict a certain clinical outcome (Fig. 5b) [28]. Each level of independent factors was assigned one score and a total score was calculated by summing up the scores in each individual. The survival probability for the individuals at 1-, 3-, and 5- year was obtained through the function conversion relationship of total scores. The calibration plot for internal validation of the nomogram showed better consistency between the predicted OS outcomes and actual observations (Fig. 5c–e). The C-index was 0.54, 0.65 and 0.66 for the TNM stage, the prognostic model and the nomogram (95% CI 0.58–0.73), further indicating that our nomogram had a higher predicting consistency. The AUCs of the nomogram at 1-, 3- and 5- year OS were 0.672, 0.684 and 0.675, which were better than the models with single independent factors (Fig. 5f–h). The DCA was used to evaluate guiding significance of these models for clinical application and the results showed that the combined model was the best for predicting the OS (Fig. 5i–k). For the hypoxia-related signature, methylation-driven prognostic model and three-gene prognostic model, the C-index reached 070, 0.64 and 0.64 in TCGA database and 0.74, 0.65 and 0.65 in ICGC database, indicating a more sensitive and valuable predictive performance of hypoxia-related model.

### Evaluation of the hypoxia-related genes for predicting the recurrence of HCC patients

**Fig. 6:**
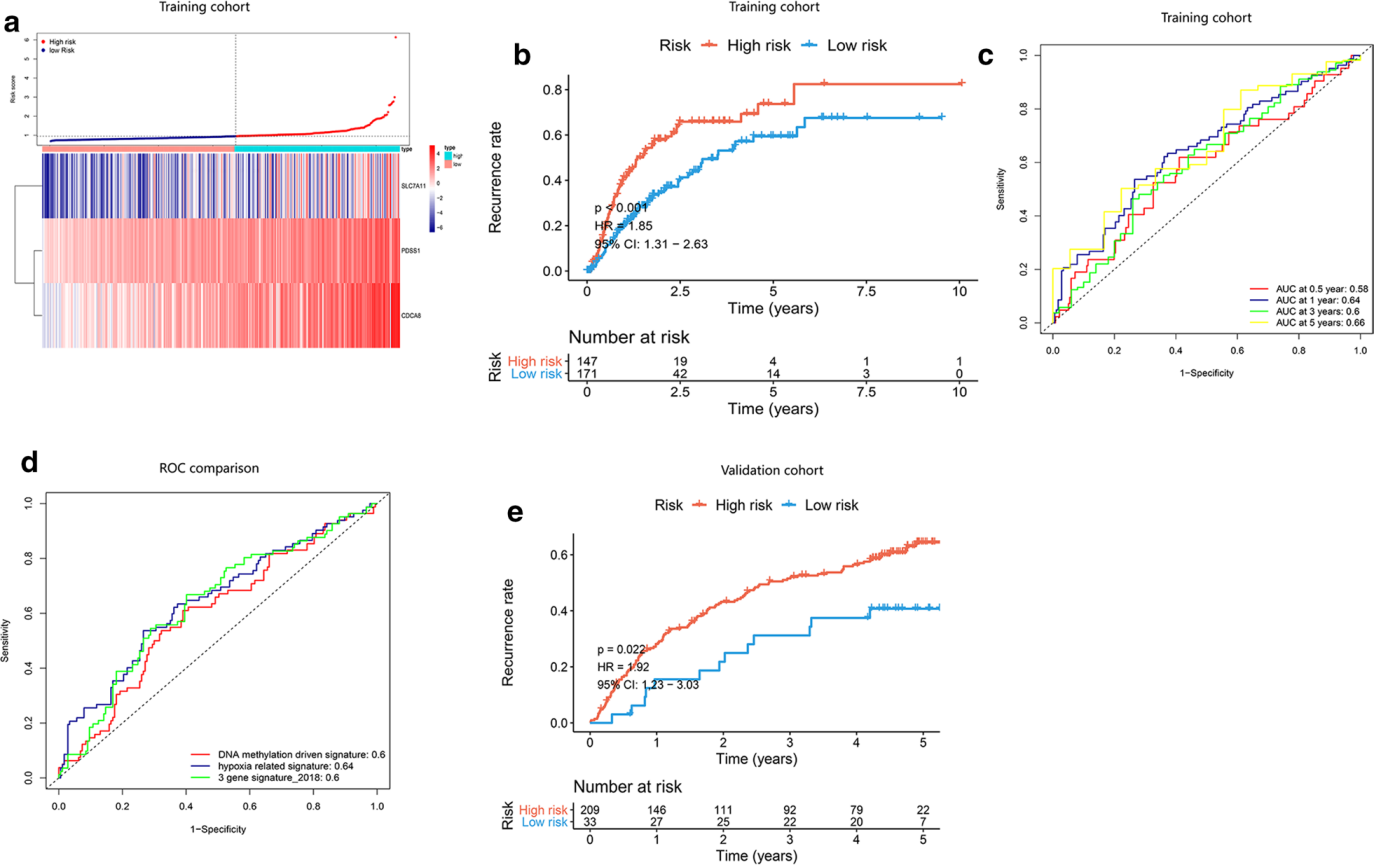
Kaplan–Meier analysis, risk score analysis, time‐dependent ROC analysis for the recurrence model based on three-gene signature in HCC. **a** Distribution of risk scores of HCC patients with different gene expression levels in TCGA cohort. **b** The recurrence rate of high- and low-risk group in TCGA. **c** Time‐dependent ROC analysis for recurrence prediction in TCGA cohort. **d** The ROC comparation between hypoxia-related recurrence signature and other recurrence models

TCGA-LIHC cohort with release-free survival (RFS) information and recurrent status of HCC patients was utilized as a training set for an independent evaluation, and the HCC cohort from GSE14520 (Additional file 8: Table S6) was used as a validation set. Based on these three hypoxia-related genes, we constructed a recurrence signature by using the regression coefficient (β’) of multivariate Cox ccproportional hazards. The prognostic index (PI) = (0.060 * expression level of PDSS1) + (0.045* expression level of SLC7A11) + (0.041* expression level of CDCA8). In both training and validation set, patients were divided into a high- and low-risk group based on the risk score of 0.953 and 1.247. The distribution of risk score and gene expression was examined (Fig. 6a, Additional file 9: Fig. S3A). From the results of Kaplan–Meier survival analysis, patients in high-risk group had significantly higher recurrence rate than the low-risk group. (Fig. 6b, e) and we also performed ROC analysis to evaluate the predictive accuracy of our recurrence model (Fig. 6c, Additional file 9: Fig. S3B). Compared with other prognostic models, the AUCs was 0.64, 0.6 and 0.6 for the hypoxia-related signature, methylation-driven prognostic model and three-gene prognostic model (Fig. 6d). All these results indicated a more reliable predictive ability of our hypoxia-related recurrence model.

### Building a nomogram for predicting recurrent probability of HCC patients and evaluating its predictive performance

**Fig. 7:**
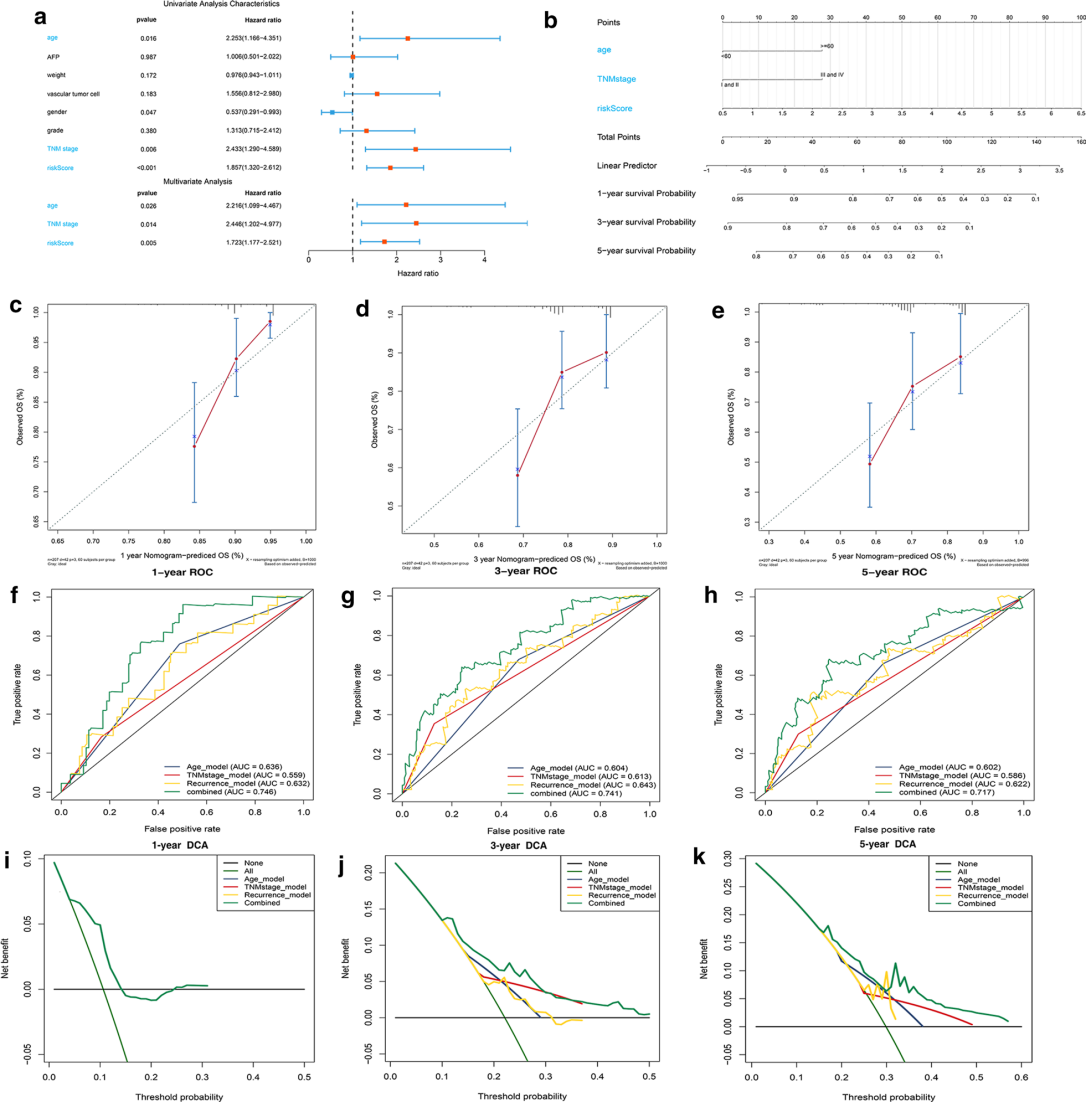
Construction of a recurrence nomogram for HCC patients in the TCGA cohort. **a** Forrest plot of the univariate and multivariate association of the risk-score model and clinicopathological characteristics with overall survival. **b** The nomogram was built based on three independent prognostic factors for predicting the recurrence in HCC patients at 1-, 3-, and 5-year. **c**–**e** The calibration plot for internal validation of the nomogram. **f**–**h** Time‐dependent ROC curves of the nomogram for 1‐,3‐ and 5‐year recurrence prediction in HCC to evaluate the predictive performance of the nomogram. **i**–**k** DCA curves of the nomogram for 1‐,3‐ and 5‐year recurrence prediction in HCC to evaluate the clinical decision-making benefits of the nomogram

We performed a univariate and multivariate Cox regression analysis and screened out three independent factors related to the recurrence of HCC (P < 0.05) (including the age, the TNM stage and the risk score of our recurrence signature) (Fig. 7a). The nomogram for recurrence prediction was built by integrating these three factors (Fig. 7b) The level of each factor was assigned according to the regression coefficient of each influencing factor, and then the scores were added to obtain the total score. Finally, the predicted value of the individual outcome was calculated through the function conversion relationship between the total score and the probability of occurrence of outcome. The calibration plot of the nomogram showed a consistency between the prediction and observation (Fig. 7c–e). The C-index was 0.62, 0.56, 0.63 and 0.71 for the age, TNM stage, the prognostic model and the nomogram (95% CI 0.64–0.78). From the results of ROC analysis in Fig. 7f–h, the AUCs of nomogram at 1-, 3-, 5-year was 0.746, 0.741, 0.717, respectively, which was obviously higher than other models with single independent factors. The DCA curves showed that the combined model obtained a higher net benefit (Fig. 7i–k). Through comparative analysis with other recurrence models, the C-index was 060, 0.59 and 0.59 for the hypoxia-related signature, methylation-driven prognostic model and three-gene prognostic model. These results indicated that our recurrent nomogram performed a better sensitivity and specificity of HCC recurrence prediction and could provide clinicians with more specific guidelines.

### Establishment of a diagnostic model based on hypoxia-related genes in HCC

**Fig. 8:**
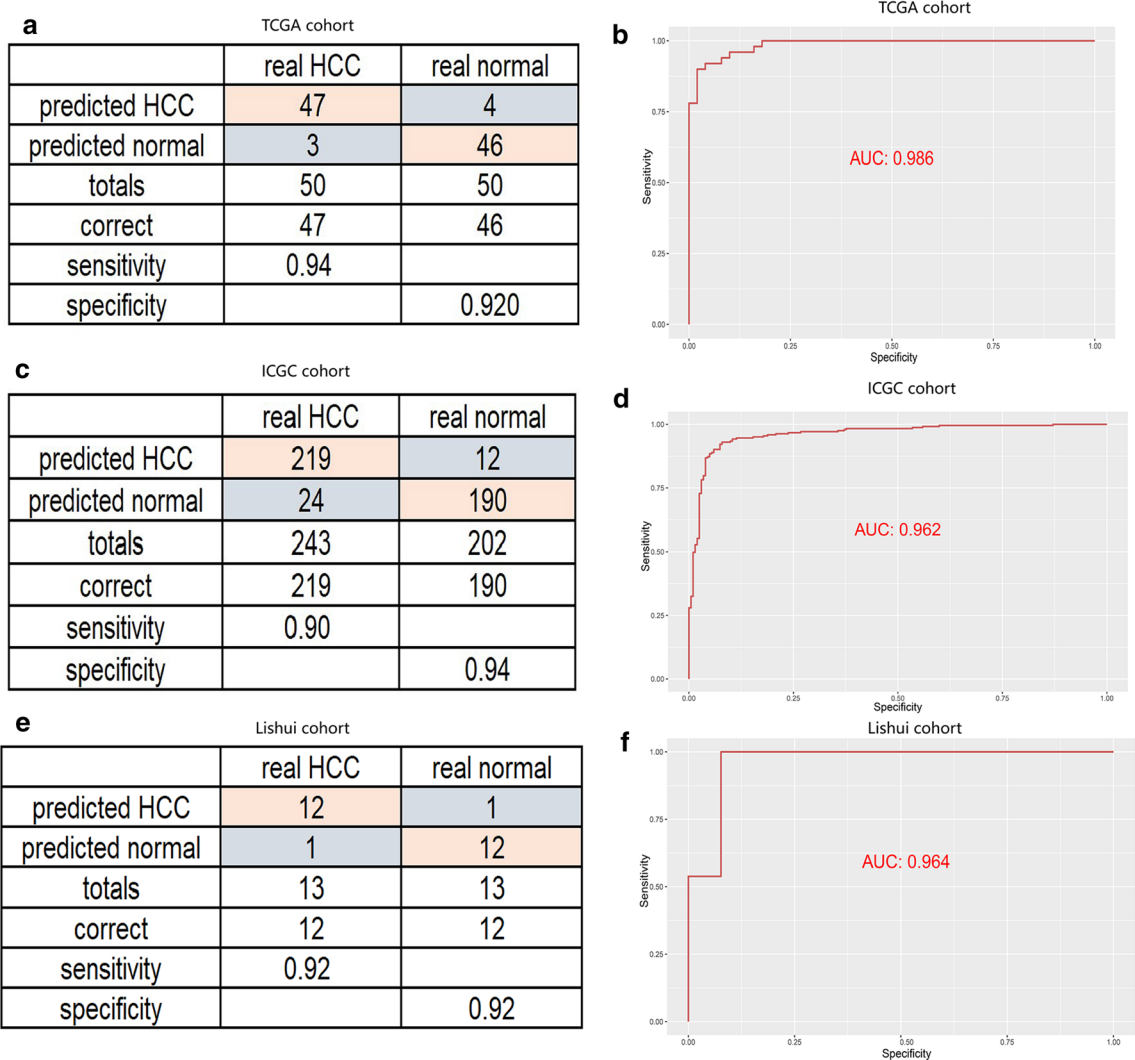
Building a diagnostic model for distinguishing HCC from normal samples. Sensitivity and specificity validation of the diagnostic model and ROC curves for evaluating the predictive performance. **a**, **b** TCGA cohort. **c**, **d** ICGC cohort. **e**, **f** in clinical specimens

As the diagnosis is of great importance for proper management of patients, we further analyzed whether hypoxia-related genes also contribute to more accurate diagnosis of HCC. A diagnostic model based on these three hypoxia-related genes was constructed by using a stepwise logistic regression method. The diagnostic score was finally identified as follows: logit (P = HCC) = 1.171 + (− 0.571) × PDSS1 expression level + (− 1.019) × SLC7A11 expression level + (− 2.037) × CDCA8 expression level. In TCGA cohort with 50 normal samples paired 50 HCC samples, our diagnostic model achieved a sensitivity of 94% and a specificity of 92% (Fig. 8a). We also utilized ICGC cohort with 190 normal samples paired 219 HCC samples as a validation set, and the diagnostic model obtained a sensitivity of 90% and a specificity of 94% (Fig. 8c). As shown in ROC analysis (Fig. 8b, d), the AUCs of our model reached 0.986 and 0.962 in TCGA and ICGC cohort, indicating a satisfactory accuracy of prediction. To further verify the clinical application of the model, we collected a group of patient-derived tissues, in which 13 tumor tissues were paired with 13 adjacent tissues. The results were proved to be satisfactory as a sensitivity of 92% and a specificity of 92% were calculated. (Fig. 8e, f).

**Fig. 9:**
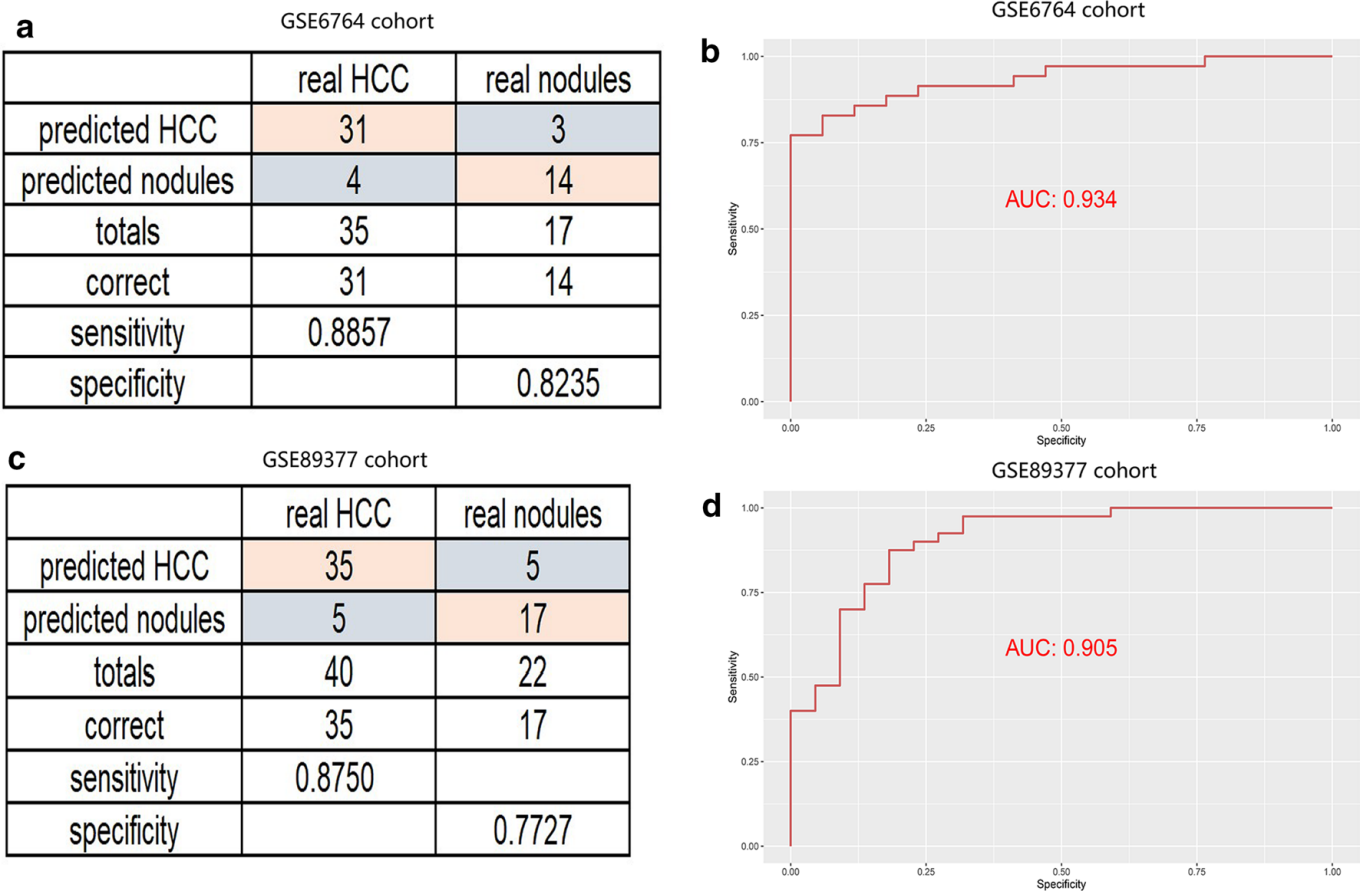
Building a diagnostic model for distinguishing HCC from dysplastic nodules. **a**–**c** sensitivity and specificity validation of the diagnostic model in the GSE6764 and GSE89377 cohort. **b**, **d** ROC curves for evaluating the predictive performance of the diagnostic model

Liver nodule is a kind of hepatic hyperplasia caused by various factors. It is indistinguishable from the early stage of liver cancer, and the corresponding treatment methods are different. We aimed to establish a diagnostic model by using a stepwise logistic regression method to better distinguish liver cancer from hepatic nodules. The diagnostic score was identified as follows: logit (P = HCC) =  − 45.308 + 0.628 × PDSS1 expression level + 8.452 × SLC7A11 expression level + 4.047 × CDCA8 expression level. We tested the diagnostic performance of the model in two databases, GSE6764 and GSE89377 cohort. One achieved a sensitivity of 88.57% and a specificity of 82.35%, the other one achieved a sensitivity of 87.5% and a specificity of 77.27% (Fig. 9a, c). The AUCs for GSE6764 and GSE89377 were 0.934 and 0.935 (Fig. 9b, d). These data further confirmed that the diagnostic model was a novel predictive tool with high accuracy and potential clinical value.

### Validation of the expression and genetic alterations and independent prognostic analysis for genes

**Fig. 10:**
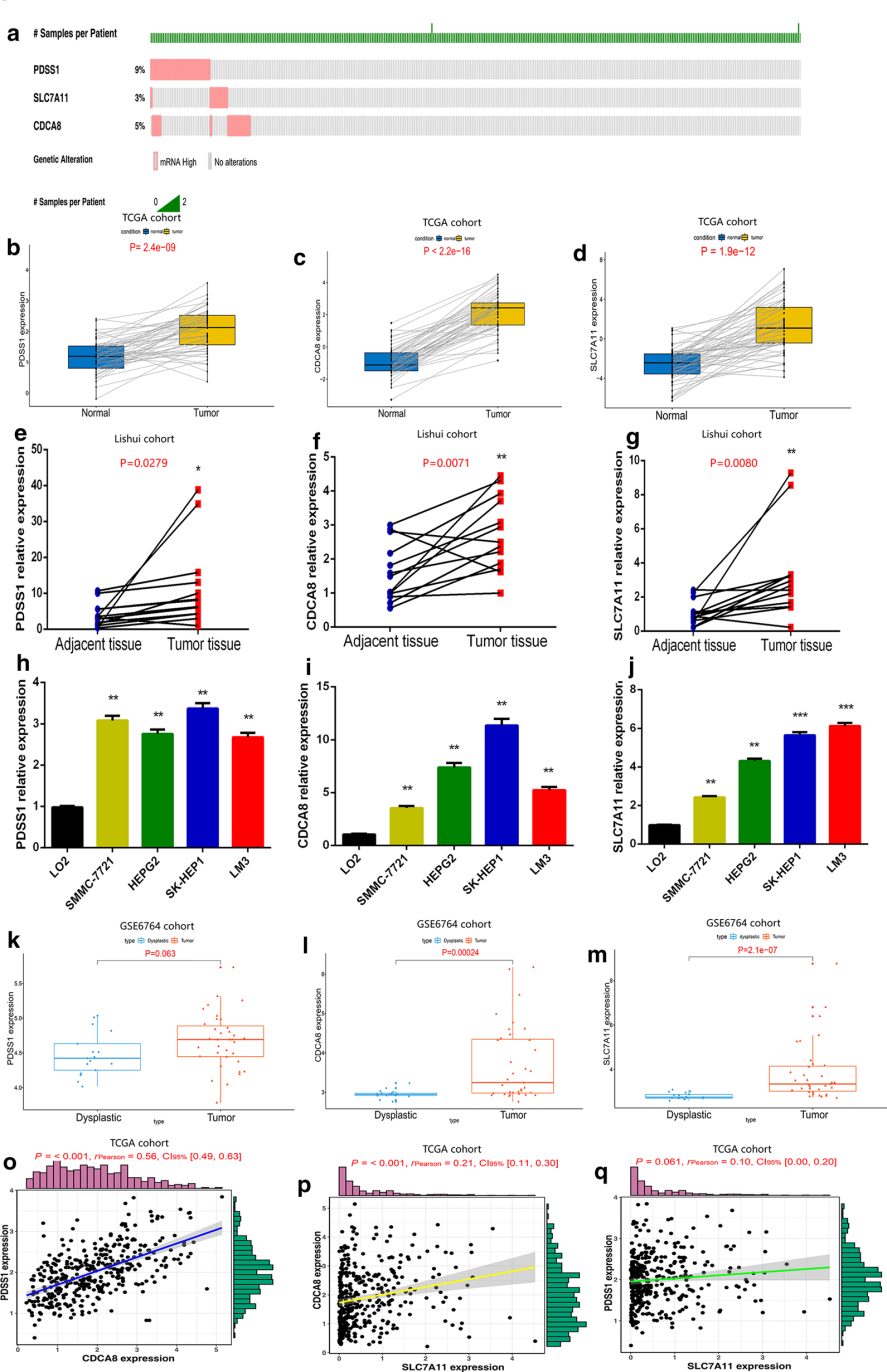
Validation of the expression characteristics of hypoxia-related genes. **a** Genetic alteration detection of the hypoxia-related genes from the cBioPortal database. **b**–**d** The expression level of each gene in TCGA cohort with 50 HCC samples paired 50 normal samples. **e**–**g** Real-time PCR analyses of the mRNA expressions of each gene in clinical specimens. **h**–**j** Real-time PCR analyses of the mRNA expressions of each gene in different cell lines. **k**–**m** The expression level of each gene in GSE6764 cohort with 35 HCC samples paired 17 dysplastic nodules. **o**–**q** The correlation analysis between expression levels of different genes in TCGA cohort with 370 HCC samples

We detected genetic alterations of the three genes from cBioportal database [29] and found that PDSS1, SLC7A11 and CDCA8 possessed genetic alterations of 9%, 3% and 5% (Fig. 10a). These results helped explain that the abnormal gene expression may be attributable to genetic alterations. To further confirm the expression level of each gene in HCC, we used TCGA database containing 50 tumor and 50 normal samples. We found all the three genes were highly expressed in HCC compared with in normal liver tissues (Fig. 10b–d). The assessment of mRNA expression for each gene in HCC clinical specimens shows that it is higher in tumor tissues (P < 0.05) (Fig. 10e–g). The protein expression of CDCA8 and SLC7A11 (also known as xCT) by IHC were showed in Additional file 10: Figure S4. We also detected the mRNA expression of these three genes in normal hepatocytes and different hepatoma cell lines. The results showed that PDSS1, SLC7A11 and CDCA8 were significantly upregulated in hepatoma cell lines (P < 0.05) (Fig. 10h–j). Moreover, by analyzing gene expression in GSE6764 cohort, we found that the expression levels of PDSS1, CDCA8 and SLC7A11 were significantly higher in tumor tissue than those in liver nodules (Fig. 10k–m). We also attempted to explore the interaction between each two genes. As shown in Fig. 10o–q, there was a sort of synergy between CDCA8 and PDSS1 as well as SLC7A11 (P < 0.05).

**Fig. 11:**
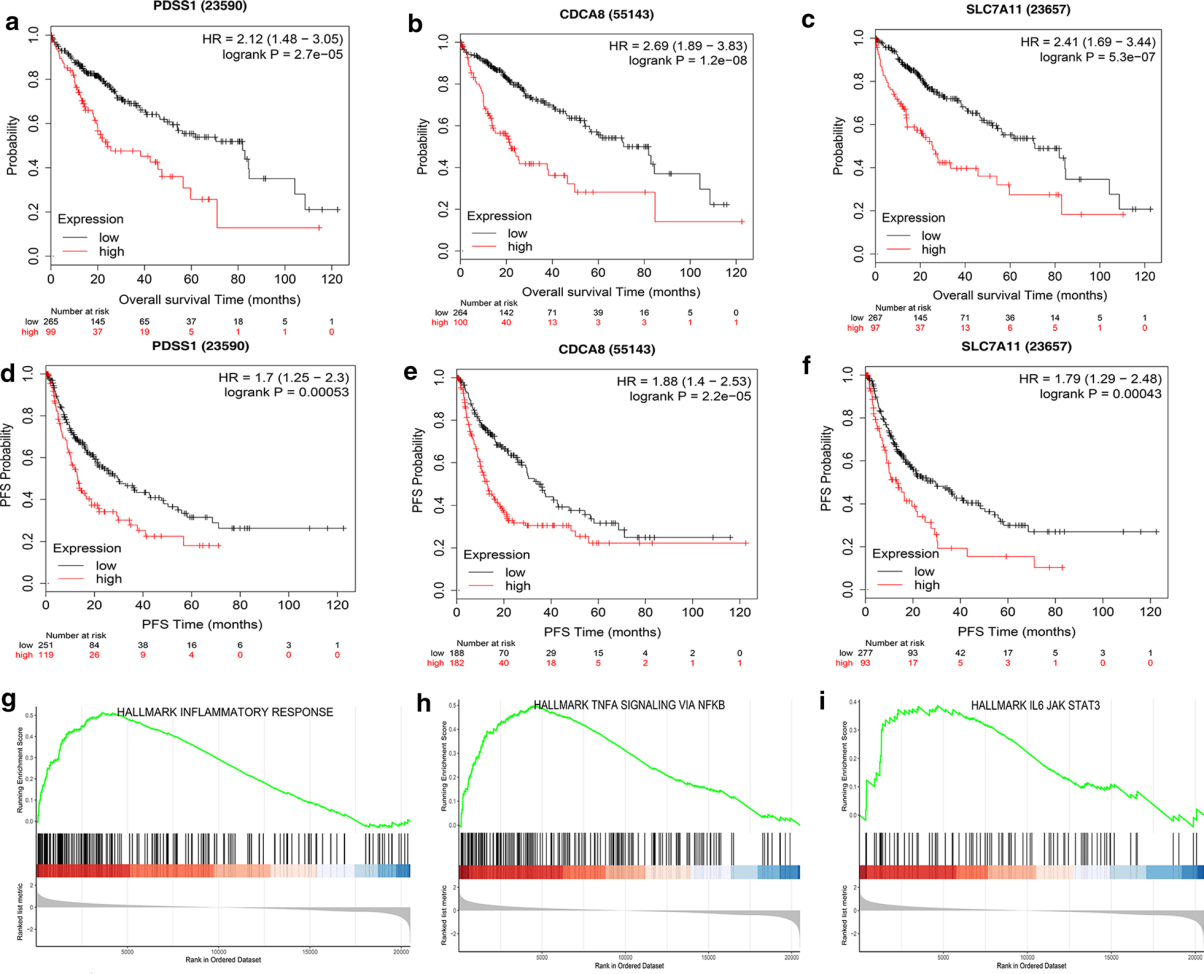
Prediction performance of hypoxia-related genes for OS and Gene Set Enrichment Analyses of the three-gene signature. **a**–**c** K–M survival curves for high and low expression levels of each gene. **d**–**f** progression-free survival analysis for high and low expression levels of each gene. **g**–**i** Three representative Kyoto Encyclopedia of Genes and Genomes (KEGG) pathways in high-risk group via GSEA

Kaplan–Meier Plotter database [30] was used in order to analyze the effect of single gene on HCC prognosis. The results showed that the high‐expression level of PDSS1, CDCA8 or SLC7A11 was separately related to a shorter overall survival time (Fig. 11a–c). In addition, the progression-free survival (PFS) analysis, which can better reflect tumor progression and predict clinical benefits, also showed an association between higher expression level of a single gene and faster disease progression (Fig. 11d–f). To achieve a better understanding of the functional characteristics of three genes, we performed Gene set enrichment analysis, which showed that some immune-related pathways, such as JAK–STAT3 signaling, The NF-kappa B signaling, were highly active in the high-risk group (Fig. 11g–i).

### Comparison of the immune microenvironment between high- and low-risk groups

**Fig. 12:**
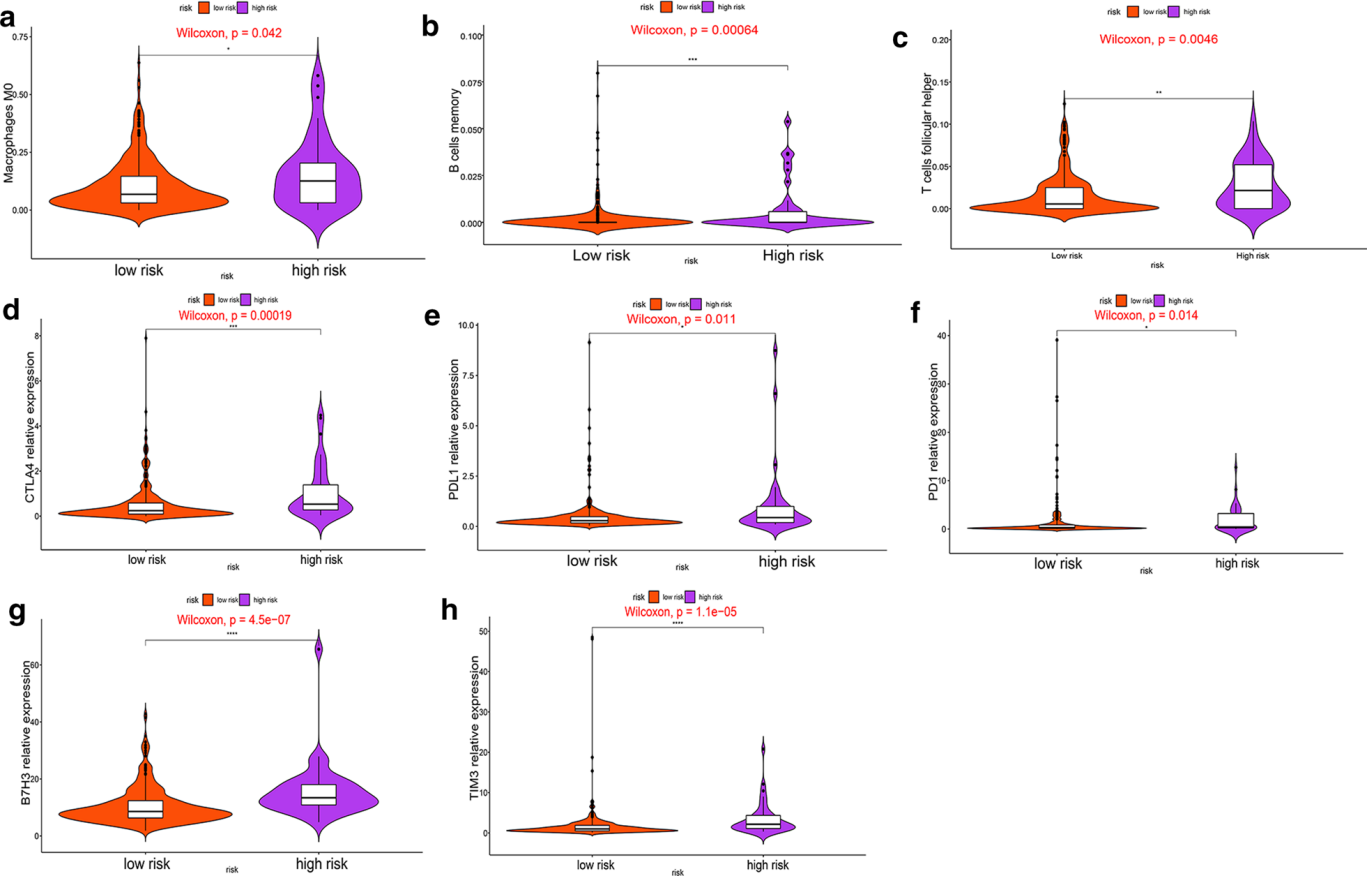
The overview of immune infiltration and expression of immune checkpoints in HCC patients with different risk scores. **a**–**c** Violin plots showing infiltration fractions of different immune cells in the high- and low-risk groups. **d**–**h** Violin plots showing the expression level of immunocheckpoints in high- and low-risk groups

Tumor immune cell infiltration refers that the immune cells move from the blood to the tumor tissue. The immune cells in tumors are closely related to clinical outcomes and they are most likely to serve as drug targets to improve survival rate [31]. Since these three genes have been found to enriched in some immune pathways, we then analyzed the relationship between hypoxia-related genes and immune cell infiltration as well as immune checkpoints in HCC. Patients in the high-risk group had higher ratios of M0 macrophages, memory B cells and follicular helper T cells than those in the low-risk group (P < 0.05) (Fig. 12a–c). Moreover, we found that the expression levels of TIM3, B7H3, CTLA4, PD1 and PDL1 in the high-risk group were obviously higher than those in the low-risk group (P < 0.05) (Fig. 12d–h). Our findings lead us to conclude that tumor immune microenvironment may be responsible for the prognosis of HCC patients with high expression of hypoxia-related genes.

## Discussion

Hepatocellular carcinoma (HCC) is one of the leading causes of cancer-related death in the world, and the development of HCC is a complicated process influenced by various factors [32]. Though some progresses have been made in the treatments of HCC, such as surgical resection, microwave ablation and liver transplantation, the prognosis of HCC patients remains poor [33]. In recent years, high-throughput sequencing and data analysis have gradually become more significant tools for biomedical research, which can identify biomarkers for prognosis predicting, recurrence monitoring as well as clinical stratification [3, 34, 35]. Therefore, it is urgent to apply to HCC and explore key targets for the treatment.

Hypoxia is a prominent characteristic of malignant tumors, especially in HCC [36]. It was demonstrated in several studies that hypoxia was involved in the aggressive development of HCC [8]. Nevertheless, due to the multiple roles of hypoxia, the specific role of hypoxia in the development of liver cancer remains unclear [37]. In this study, we identified three hypoxia-related genes (PDSS1, CDCA8 and SLC7A11) closely relating to HCC. CDCA8, involving in protein metabolism and mitosis, has been demonstrated to participate in malignant progression of tumor cells and lead to poor prognosis in liver, stomach and lung cancer [38]. SLC7A11 (also known as xCT) plays a critical role in maintaining redox homeostasis and has been confirmed to be associated with the prognosis of HCC [39]. PDSS1 is involved in coenzyme Q biosynthesis, but little is known about the relationship between PDSSI and cancer [40]. Our results showed that this three-gene signature was an independent factor affecting the prognosis of HCC and the model had a better predictive performance on both prognosis and recurrence. What’s more, the diagnostic model based on these three genes had a high sensitivity and specificity, and could help distinguish HCC from dysplastic nodules. Consensus Clustering is a common method for classification of cancer subtypes. We divided the samples into 4 clusters according to the hypoxia-related DEGs dataset of HCC and compared the differences among clusters. It should be pointed out that cluster-2 had a higher TMB, indicating that patients in cluster-2 were more likely to benefit from immunocheckpoint inhibitor therapy [41].

Much work so far has focused on the role of hypoxia in regulating the immune response in tumors. Hypoxia can interfere with the differentiation and function of immune cells through regulating the expression of co-stimulating receptors and the types of cytokines [42, 43]. The immune system is able to recognize and eliminate tumor cells through innate and adaptive mechanisms. However, the tumor microenvironment could suppress this anti-tumor response through a number of inhibitory pathways which were known as immunocheckpoints [44]. Our results of GSEA indicated that hypoxia-related signature could positively regulate some immune signaling pathways. The high-risk group based on the expression level of hypoxia-related genes had a higher infiltration proportion of macrophages, B memory cells and follicle-assisted T cell, as well as higher expression levels of immune checkpoints. These evidence for the association between hypoxia and immunity highlighted the importance of immunotherapy for HCC patients with high expression level of three hypoxia-related genes.

However, some limitations of this study should be noted. First, the process of adjusting the weight of regression coefficient in LASSO might ignore some important factors contributing to HCC prognosis. Second, our nomogram did not perform external validation as there was a lack of specific clinical data in ICGC database. Moreover, our retrospective findings need to be further validated in prospective research. Finally, the complex interaction between tumor cells and immune cells in hypoxic environments remains to be further explored.

## Conclusion

In summary, we identified the hypoxia-related DEGs between HCC and normal tissues and clustered HCC samples into 4 subgroups. We established the diagnosis, prognosis and recurrence models based on three hypoxia-related genes, which performed favorable diagnosis and prediction performance for HCC. Finally, we identified higher proportions of immune cell infiltration and immunocheckpoint expression in the high-risk group, which may be more sensitive to benefit from immunotherapy.

## Supplementary information

**Additional file 1:** Table S1. Hypoxia-related gene expression profile of HCC in TCGA-LIHC.

**Additional file 2:** Table S2. The mRNA-sequencing data of HepG2 cells exposed to normoxia and hypoxia for 24 hours in GSE41666 dataset.

**Additional file 3:** Table S3. The mRNA-sequencing data in of Huh-7 cells under normoxia and hypoxia for 24 hours in GSE59729 dataset.

**Additional file 4:** Table S4. Univariate analysis with Cox proportional hazard model.

**Additional file 5:** Figure S1. Identification of key hypoxia-related genes closely related to the prognosis of HCC. **A**–**B** LASSO-penalized Cox regression. The dataset was subsampled 1000 times and chose the genes repeated >900 times.

**Additional file 6:** Table S5. Hypoxia-related gene expression profile of HCC in ICGC cohort.

**Additional file 7:** Figure S2. The ROC comparation between hypoxia-related prognostic signature and other published prognostic models. **A** in TCGA database. **B** in ICGC database.

**Additional file 8:** Table S6. Hypoxia-related gene expression profile of HCC in GSE14520 cohort.

**Additional file 9:** Figure S3. Time-dependent ROC analysis for the recurrence model based on hypoxia-gene signature in HCC. **A** Distribution of risk scores of HCC patients with different gene expression levels in ICGC cohort. **B** Time-dependent ROC analysis for recurrence prediction in ICGC cohort.

**Additional file 10:** Figure S4. Representative images of CDCA8 and xCT immunohistochemistry in HCC tissues.

## Data Availability

The data used to support the findings of this study are available from the corresponding author upon request.
