# Supplementary figures and images for: A hypoxia-related signature for clinically predicting diagnosis, prognosis and immune microenvironment of hepatocellular carcinoma patients
Source: J Transl Med. 2020 Sep 4;18:342. doi: 10.1186/s12967-020-02492-9 (PMC7487492; doi:10.1186/s12967-020-02492-9)

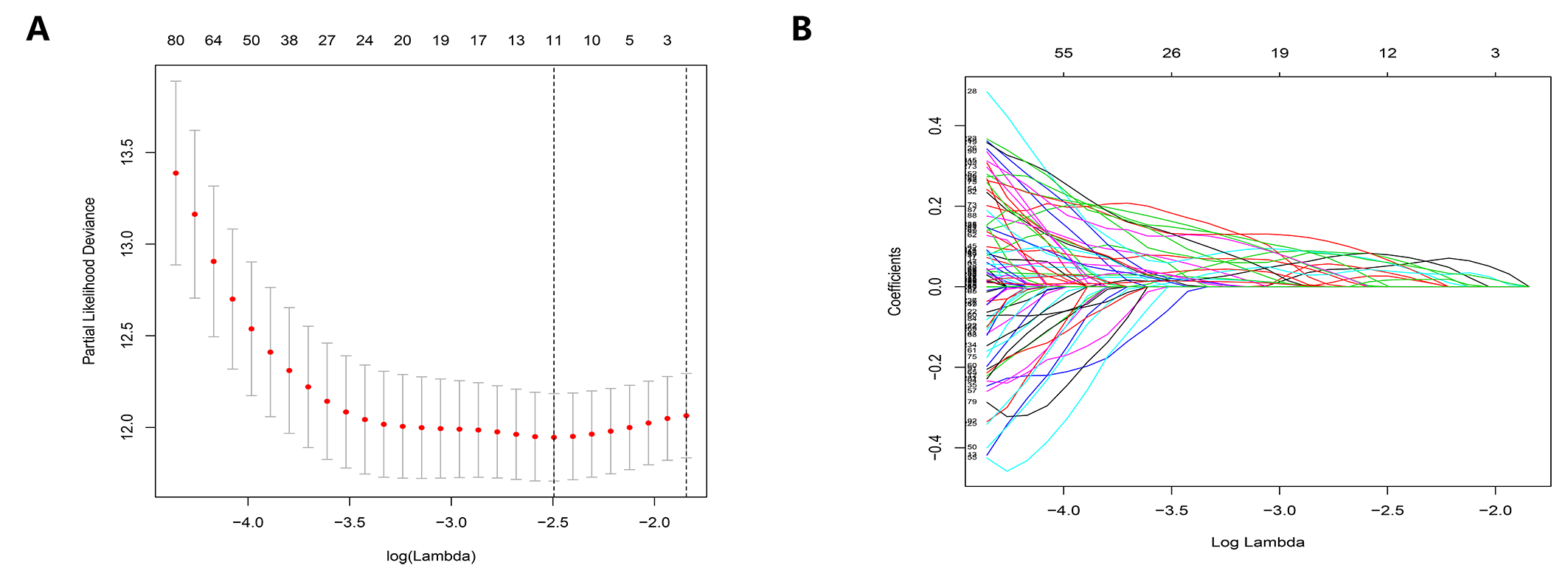

Supplement: Supplementary file 5 — Additional file 5: Figure S1. Identification of key hypoxia-related genes closely related to the prognosis of HCC. A–B LASSO-penalized Cox regression. The dataset was subsampled 1000 times and chose the genes repeated >900 times. [file 12967_2020_2492_MOESM5_ESM.tif]

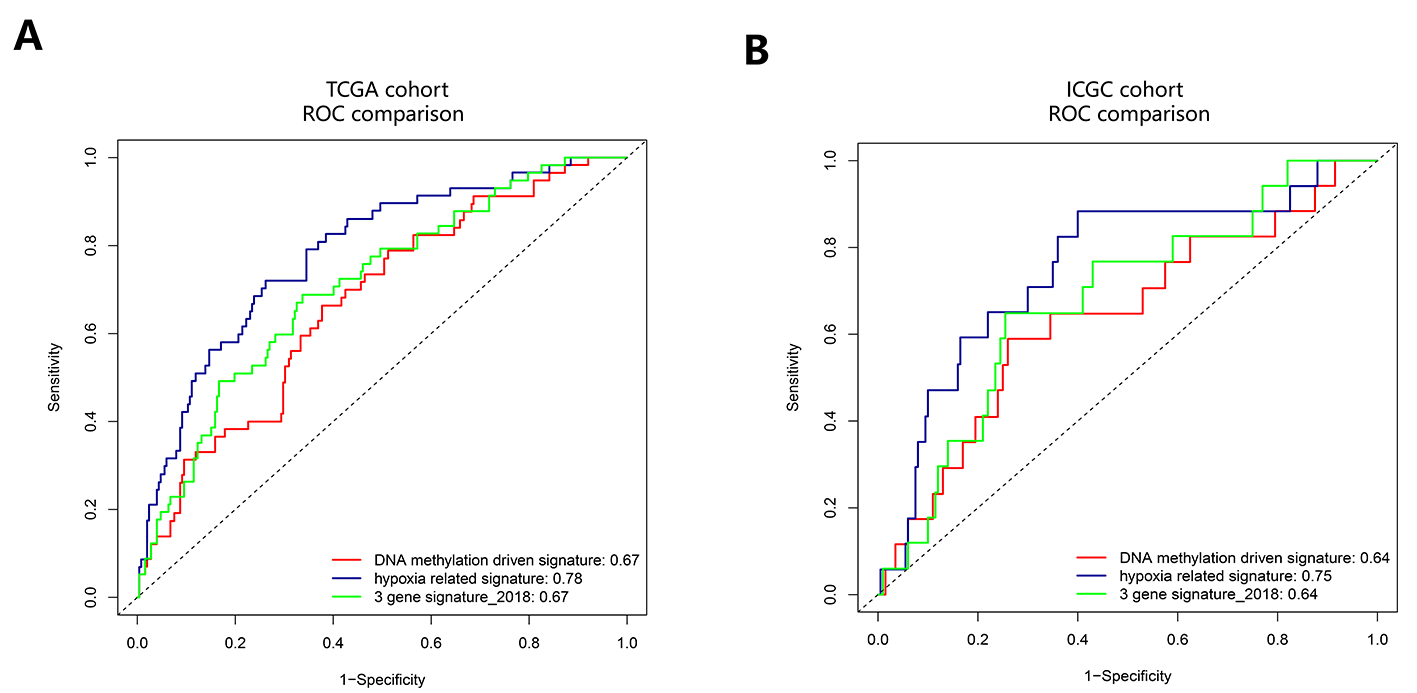

Supplement: Supplementary file 7 — Additional file 7: Figure S2. The ROC comparation between hypoxia-related prognostic signature and other published prognostic models. A in TCGA database. B in ICGC database. [file 12967_2020_2492_MOESM7_ESM.tif]

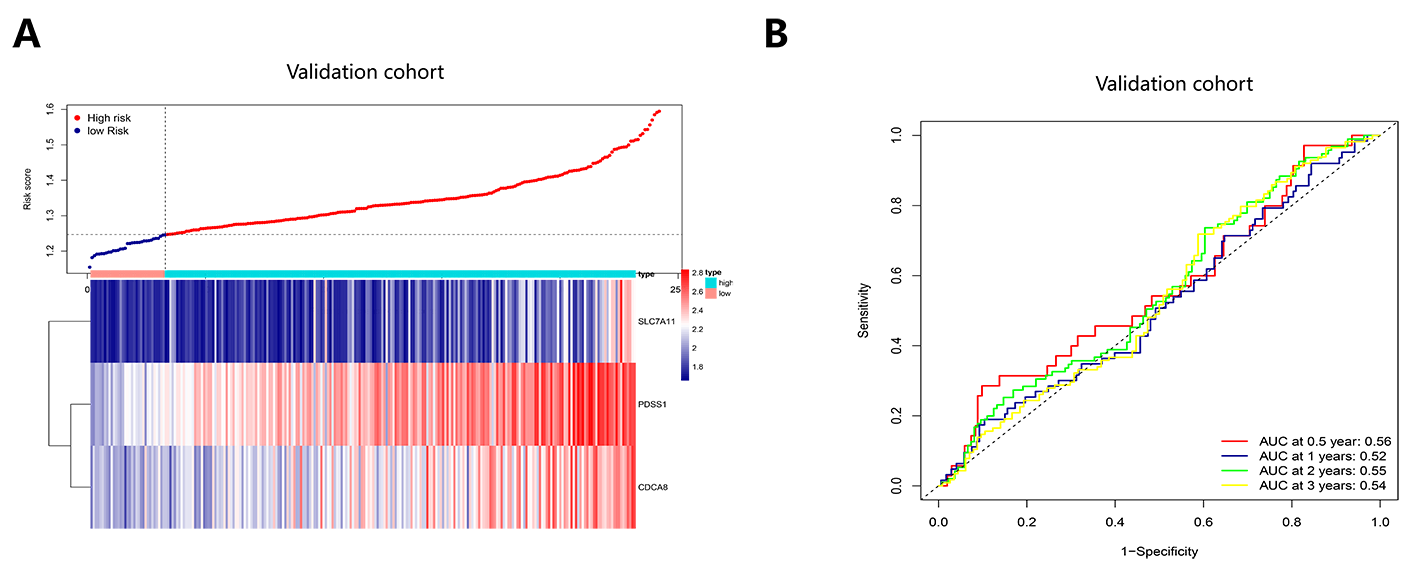

Supplement: Supplementary file 9 — Additional file 9: Figure S3. Time-dependent ROC analysis for the recurrence model based on hypoxia-gene signature in HCC. A Distribution of risk scores of HCC patients with different gene expression levels in ICGC cohort. B Time-dependent ROC analysis for recurrence prediction in ICGC cohort. [file 12967_2020_2492_MOESM9_ESM.tif]

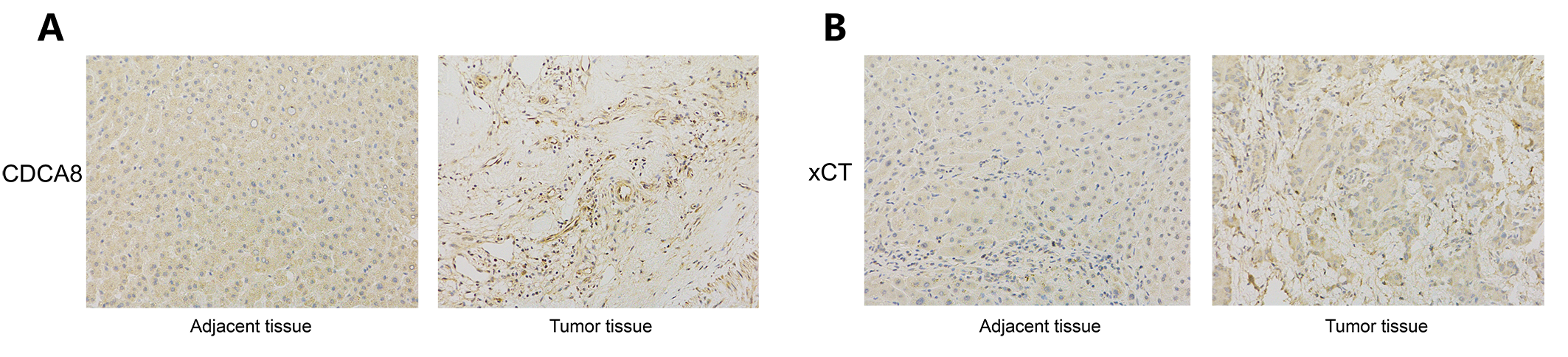

Supplement: Supplementary file 10 — Additional file 10: Figure S4. Representative images of CDCA8 and xCT immunohistochemistry in HCC tissues. [file 12967_2020_2492_MOESM10_ESM.tif]
